# Operating status of public toilets in the Hutong neighborhoods of Beijing: An empirical study

**DOI:** 10.1016/j.jenvman.2021.112252

**Published:** 2021-06-01

**Authors:** Rui Yan, Shikun Cheng, Jingang Chen, Xiangkai Li, Sumit Sharma, Sayed Mohammad Nazim Uddin, Heinz-Peter Mang, Cong Chen, Zifu Li, Tianxin Li, Xuemei Wang

**Affiliations:** a School of Energy and Environmental Engineering, Beijing Key Laboratory of Resource-oriented Treatment of Industrial Pollutants, University of Science and Technology Beijing, Xueyuan Road No.30, Haidian District, Beijing, 100083, PR China; b Cologne University of Applied Science, Hahnen Str. 31c, 50354, Huerth-Efferen, Germany; c Environmental Science Program, Asian University for Women, 20/A, M M Ali Road, Chittagong, Bangladesh; d German Toilet Organization, Paulsenstr. 23/12163, Berlin, Germany; e School of Economics and Management, University of Science and Technology Beijing, Xueyuan Road No.30, Haidian District, Beijing, 100083, PR China

**Keywords:** Public toilet, Fault tree analysis, Delphi, Fecal sludge, Hutong, Non-sewered

## Abstract

The provision of sanitation services for fast-growing urban populations is one of the world's urgent challenges. Hutong neighborhoods in Beijing, capital of China, cannot be rebuilt due to the protection of historical heritage, while residents still need to keep the habit of defecating in public toilets. One hundred public toilets with non-sewered sanitation in the Hutong neighborhoods of Beijing were visited to investigate the actual operating status in response to the “toilet revolution” campaign. The fault tree approach was used to identify the barriers toward a decent and environment-friendly public toilet and evaluate potential risks from the malfunction of various components. Four subsystems are defined and elaborated to calculate the fault possibility. These subsystems are environment- and user-friendly, regarded as ancillary facilities, and used for fecal sludge (FS) management. Statistical analysis of targeted cases indicated that fault probabilities of environmental considerations, user-friendly considerations, ancillary facilities, FS management are calculated as 0.79, 0.96, 0.96, and 0, respectively. The subsystems were weighted using a Delphi method concept. Results showed that the well operation ratio of Beijing Hutong public toilets is only 32%, and the sanitation service value chain can be further optimized. This study also provides references for other countries, which are dedicated to promoting urban sanitation and public health.

## Highlights

• Potential risks of public toilets were identified through Fault Tree Analysis.
• A methodology in framing failures of public toilet subsystems is developed.
• Delphi method was applied, validated, and found useful in sanitation research.
• The barriers toward decent and environment-friendly public toilets are identified.

## 1 Introduction

People in the world are endeavoring to ensure access to safe water sources and sanitation for all, which is an ambitious goal under United Nations Sustainable Develop Goals (SDG) (target 6). Sanitation is a matter of hygiene, health, dignity, and human rights. In 2010, the United Nations General Assembly (UNGA) explicitly recognized the human right to access to improved sanitation, and the Human Rights Council reaffirmed this recognition, even place it as a precursor to other human rights (UNGA, 2010). In 2017, 82% and 45% of the global population (6.2 billion and 3.4 billion people) worldwide used improved sanitation facilities and safely managed sanitation services, respectively (UNICEF and WHO, 2019). Shared sanitation is a double-edged sword. On the one hand, shared sanitation is treated as an interim solution and serves for a considerable number of people, especially for densely populated households in fast-growing urban areas in low income countries. One the other hand, shared sanitation is treated as transmitters of disease and likely to increase exposure to health risks (Greed, 2006). Public toilet, a type of improved sanitation facility being shared, is a vital component in urban planning to create accessible, sustainable, and livable cities for all (Greed, 2004); therefore, adequate and sanitary public toilets should be provided. However, many so-called modern and livable cities are not equipped with qualified public toilets that match their economic development and international prestige (Siu, 2006). The public toilets have a long history and most of these were constructed and managed by municipalities in the form of communal latrines (Antoniou et al., 2016). Which ancient civilization and country gave birth to the earliest public toilet is still obscured; people could reach a consensus on the development of modern public toilets, which dates from the late 19th century when the flushing toilets were widely used, the water supply was improved, and the sewer system were constructed in combination with waste management provision and public health legislation in the United Kingdom (Stanwell-Smith, 2010). In the past years, urban sanitation strategies and planning frameworks are comparatively neglected to achieve considerable sanitation improvements because investments commonly go towards visible services, such as water supplies and drainage networks (Hutchings et al., 2018; Scott et al., 2019). The model of flushing toilet access to the sewer system, which originates from western countries, has become the paradigm for the developing countries during rapid urbanization if cities are to be healthy, livable, and safe.

Beijing is expanding with urbanization in the past years. An increasing number of sewers and centralized sewage treatment facilities were constructed. In 2018, the total sewage pipeline has reach 12,147 km in length. The sewage treatment capacity of Beijing has reached 6.706 million m^3^/day (1.903 billion m^3^/year) with a sewage treatment rate of 93.4%.

Beijing, the capital of China and the megacity in the world, is well-known for its time-honored histories and cultures. Hutong,^1^ which originated from Yuan Dynasty, is the historic grid pattern of residential lanes, which are examples of ancient Chinese city planning. Most buildings existing in Hutong today were originally built during the early Qing period of the mid-seventeenth century (Hou, 2000). The Hutong neighborhoods are clusters of one-story residential buildings, which are composed of private courtyards, also known as siheyuan. Siheyuan is a quadrangle dwelling with the main building located on the northern side and two subrooms on the western and eastern sides; meanwhile, the south side has a building facing the street and a main gate (Steinhardt, 2002). The residents in Hutong neighborhoods use public toilets, rather than private ones at home. Hutong, regarded as the feature of old Beijing urban architecture, has witnessed the historical changes of Beijing; thus, Hutong is a historical and cultural heritage of Beijing. In 2003, 658 courtyard dwellings (siheyuan) were selected from thousands of candidates for protection (Chen and Zhang, 2019). Hutong neighborhoods should be kept intact without being reconstructed. In such a case, infrastructure cannot be constructed, which means that Hutong neighborhoods cannot be connected to the sewer and municipal pipeline. Residents must keep the habit of defecating in public toilets.

**Fig. 1:**
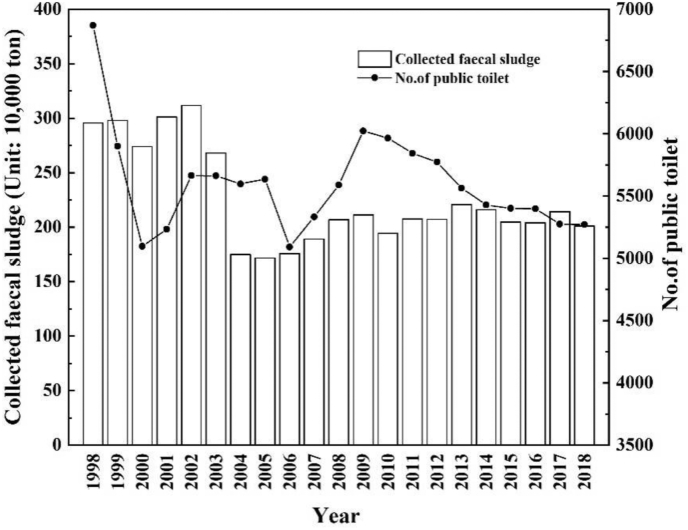
Quantity of public toilet and collected fecal sludge (FS) in urban Beijing.

Public toilet is a mirror and a barometer, which can reflect the civilization of a city, even a nation. Since 2015, “toilet revolution” has become a major topic, and a nationwide campaign has been launched in China to promote sanitation coverage and people's wellbeing in every aspect. Beijing, the capital of China, had retrofitted 804, 1145, and 580 sets of public toilets in 2016, 2017, and 2018, respectively. In 2018, 5270 sets of public toilets were recorded in the urban areas of Beijing, 5250 of which could meet the standard of Grade 3 and above (Beijing Municipal Bureau of Statistics, 2019). Fig. 1 presents the quantity of public toilet and collected FS in urban Beijing from 1998 to 2018. However, providing sanitary public toilet is only the first step of the Hutong “toilet revolution”. The second and more difficult part is making these infrastructures used and maintained in good condition and managing FS in a well-organized and sustainable way.

Studies on public toilet is not as much as other sanitation topics. Bhatt et al. (2013) visited 100 public toilets to understand and describe the state of the nonhousehold toilet facilities in the populous urban area of Nepal. Ssekamatte et al. (2019) used a cross-sectional study design to obtain data on the experiences of public toilet operators via 20 in-depth interviews to identify opportunities and barriers to effective O & M of public toilets in informal settlements in Kampala, Uganda. Peprah et al. explored public toilet use conditions, socio-demographic characteristics, opinions of public toilet customers, and possible improvements to public toilet facilities in four neighborhoods in Accra, Ghana, which is a representative country in the world with the highest reliance on shared sanitation facilities (Ssekamatte et al., 2019). Mariwah et al. (2017) explored the user satisfaction and willingness to pay for improved public toilet, which is seated on the first step on the sanitation ladder, in two low-income communities of Accra, Ghana. Njeru (2014) used a qualitative analytical approach to elaborate how the public toilet (named as “Ikotoilets”) in Nairobi are providing reasonably hygienic and safe sanitation services in the city by rethinking local technologies of shared sanitation. Siu and Wong (2013) explored the concerns of visually impaired persons in relation to public toilets and identified methods to improve the accessibility and user participation. Chang (2014) conducted field surveys and individual interviews in Yanbian Korean Autonomous Prefecture, northern China to investigate the factors that affect the culturally and environmentally sound management of various types of public squatting-type toilet. Zhang et al. (2018) proposed a network-based location model to improve the coverage of public toilets in a large metro system of Shanghai to fulfill the needs of accessibility and public health. Biss and Park used geographic information systems to map and describe the distribution of public toilets and analyze the density of public toilets in parklands or open spaces in selected major US and international cities. The calculation results showed great differences among these cities (Bliss and Park, 2019; Park and Bliss, 2019). In summary, most studies are based on field investigation to highlight the importance of mighty public health policy, adequate public toilet coverage, effective O & M mechanism, and environment-friendly waste management (Nyambe et al., 2020).

According to Beijing Municipal 13th Five-Year Urban and Rural Environmental Development Plan (BMCCA and BMCDR, 2016), the percentage of public toilet above Grade 3 should exceed 95%. No target is set for the quantity of public toilet, thereby indicating that quality overweighs quantity in terms of Beijing public toilets. This study aims to investigate the actual operating status of public toilets in the Hutong neighborhoods of Beijing and identify the gap towards a decent and sustainable pathway in urban areas. This study also aims to develop a methodology to evaluate the operating status of public toilets and provides references for other countries, which are dedicated to promoting urban sanitation and public health.

## 2 Materials and methods

### 2.1 Study area

Most Hutong neighborhoods are located in the downtown of Beijing. According to the administrative division, these neighborhoods belong to Dongcheng and Xicheng Districts, Beijing. The existing public toilets were originally constructed in the 1960s when the residents intended to move the toilet from the courtyard to the street to spare space in their own courtyard and improve the living conditions. In this way, the honey wagon can easily collect FS (Kitsuka et al., 2007). The public toilets in the downtown of Beijing were rebuilt or newly built along with big historic events for Beijing. One event is the 1990 Asian Games, which is the first large international comprehensive sports meeting held in China. The other one is the 2008 Olympic Games, which is the Olympic held for the first time in the developing countries. The third node is the “Toilet Revolution” campaign after 2015. Most public toilets in Hutong neighborhoods are not connected to a sewer system. Public toilets were randomly selected and based on a uniform geographic distribution. On hundred public toilets were visited for this study.

### 2.2 Public toilet and its subsystem

The design and construction of public toilet in urban China should follow the *CJJ 14–2016 Standard for Design of Urban Public Toilets*, which was first published in 2005 and revised in 2016. All rebuilt and newly built public toilets should meet the standard when they are accepted for check once they are finished. However, Beijing local standard *DB11/T 190–2016 Specification for Construction of Public Toilets* added lighting and other indicators and proposed high requirements for existing indicators.

A typical public toilet consists of basic sanitary fixtures (urinal and pedestal or squatting pan) and ancillary facilities. Software components are even vital and should not be neglected compared with touchable fittings. Such components include cleanliness, smell, amenity, and privacy, all of which can be judged by visual inspection. Environment-friendly considerations, such as water-saving facilities and pollution-free waste management, are also of great importance for a conservation-oriented and sustainable society. To evaluate a public toilet, it can be treated a system composed of four subsystems: environmental considerations, user-friendly considerations, ancillary facilities, and FS management. Environmental considerations can be the general defecating and urinating atmospheres and water-saving considerations. Use-friendly considerations mainly result from gender and privacy considerations. Ancillary facilities mainly include handwashing facilities and other facilities that could make defecation and urination convenient. The FS management is the back-end of public toilet and should also be carefully considered.

A collection of photos to show the public toilet and its components is presented in the supplementary material.

### 2.3 Data analysis

**Fig. 2:**
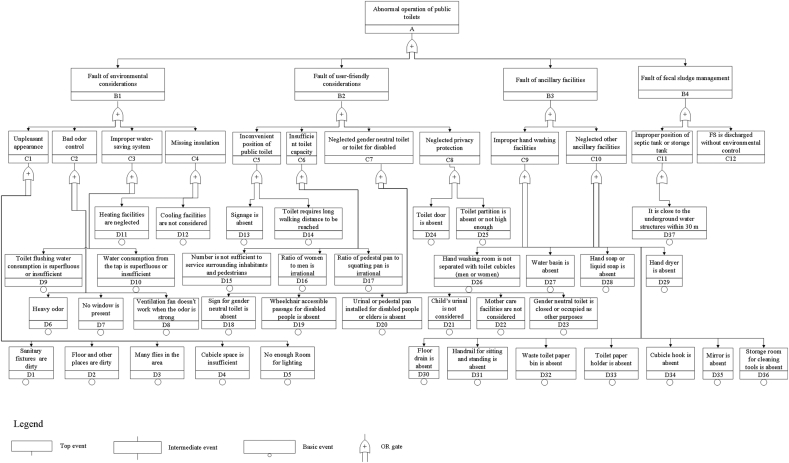
Fault tree of public toilet systems.

Park and Lee (2009) performed a fault tree analysis (FTA) of the handwashing process for hygiene management and concluded that qualitative- and quantitative-based FTA is an ideal alternative approach to hazard analysis during the implementation of hazard analysis and critical control point system. On the basis of previous research, this study employed a fault tree approach to identify potential risks and problems for a public toilet and adopted countermeasures to sustain the operation of public toilets. Fault trees with respect to public toilet are structured following the rules (Rausand and Høyland, 2003) according to the four subsystems of a public toilet system. These trees visualize faults on the basis of the operational problems observed during the field study. The top event of the fault tree is the abnormal operation of a public toilet, which is an OR gate that includes the faults of the four subsystems, namely, environmental considerations, user-friendly considerations, ancillary facilities, and FS management (Fig. 2). Each type of fault is broken down from one cause to another until the basic events responsible for the undesirable event are obtained. Every event is numbered from B1 to B4 for the first-level contributors, C1 to C12 for the second-level contributors, and D1 to D37 for the third-level contributors. Public toilet failure happens as a result of events occurring in one or more of the four subsystems (B events). Specifically, any failure in B events can result in the abnormal operation of a public toilet. Similarly, B events’ failure also results from undesirable C events until the basic events. The criteria and descriptions of failures of public toilet subsystems are presented in Table 1.

**Table 1:** Criteria and descriptions of failures of public toilet subsystems.

Subsystem	Failure criteria and descriptions	Failure assessing method
B1: Environmental considerations	C1: Unpleasant appearance: sanitary fixtures (urinal and pedestal or squatting pan), floor, and other places are dirty; the area has many flies; the cubicle space is insufficient; no enough room for lighting.	Visual inspection according to GB/T 17,217–1998 and DB11/T 356–2017
	C2: Bad odor control: the odor is heavy; no window is present; the ventilation fan does not work when the odor is strong.	Visual inspection and first-hand experience according to GB 14554–1993
	C3: Improper water consumption system: the toilet flushing water consumption is superfluous or insufficient; the water consumption from the tap is superfluous or insufficient.	Visual inspection and first-hand experience according to GB/T 31,436-2015
	C4: Missing insulation: heating facilities are neglected; cooling facilities (air conditioner or fan) are not considered.	Visual inspection and first-hand experience according to DB11/T 190–2016
B2: User-friendly considerations	C5: Inconvenient position of public toilet: the signage is absent; thus, the toilet is difficult to find; the toilet requires long walking distance to be reached.	Visual inspection; first-hand experience according to DB11/T 190–2016
	C6: Insufficient toilet capacity: the number is not sufficient to service surrounding inhabitants and pedestrians; the ratio of women to men is irrational; the queue for women toilet is long; the ratio of pedestal pan to squatting pan is irrational; thus, elders might have troubles.	Visual inspection according to DB11/T 190–2016
	C7: Neglected gender-neutral toilet or toilet for disabled: sign for gender neutral toilet is absent; wheelchair accessible passage for disabled people is absent; urinal or pedestal pan installed for disabled people or elders is absent; child's urinal is not considered; mother care facilities are not considered; gender neutral toilet is closed or occupied as other purposes.	Visual inspection according to DB11/T 190–2016 and DB11/T 356–2017
	C8: Neglected privacy protection: toilet door is absent; toilet partition is absent or not high enough.	Visual inspection according to DB11/T 190–2016
B3: Ancillary facilities	C9: Improper hand washing facilities: hand washing room is not separated with toilet cubicles (men or women); the water basin is absent; hand soap or liquid soap is absent; hand dryer is absent.	Visual inspection according to DB11/T 190–2016
	C10: Neglected other ancillary facilities: floor drain is absent; handrail for sitting and standing is absent; waste toilet paper bin is absent; toilet paper holder is absent; cubicle hook is absent; mirror is absent; storage room for cleaning tools is absent.	Visual inspection and interview according to DB11/T 190–2016
B4: FS management	C11: Improper position of septic or storage tank: it is close to the underground water structures within 30 m.	Interviews according to DB11/T 190–2016
	C12: FS is discharged without environmental control (connected to the sewer or collected for centralized treatment).	Visual inspection and interviews according to DB11/T 355–2006

Note: GB 14554–1993 is the national standard titled “Emission standards for odor pollutants”.

GB/T 17,217–1998 is the national standard titled “Hygienic standard for urban public toilet”.

GB/T 31,436–2015 is the national standard titled “Water saving sanitary ware”.

DB11/T 190–2016 is the Beijing local standard titled “Specification for construction of public toilets”.

DB11/T 356–2017 is the Beijing local standard titled “Standard for operation and management of public toilets”.

DB11/T 355–2006 is the Beijing local standard titled “Code for night-soil collecting and transporting management”.

This study is based on observation and interview in combination with qualitative and quantitative analysis. Three investigators inspected the public toilet together and presented the score of event C according to their individual observation. If the average score from three investigators is less than 60 out of 100, then it is considered as a fault event. If the average score is more than 90 out of 100, then it is considered to be in good condition. The weight of the first-level contributor (B events) is decided by ten experts according to the Delphi method. The weight of the second-level contributor (C events) is considered to be equal under their respective up-levels (B events).

## 3 Results and discussions

**Fig. 3:**
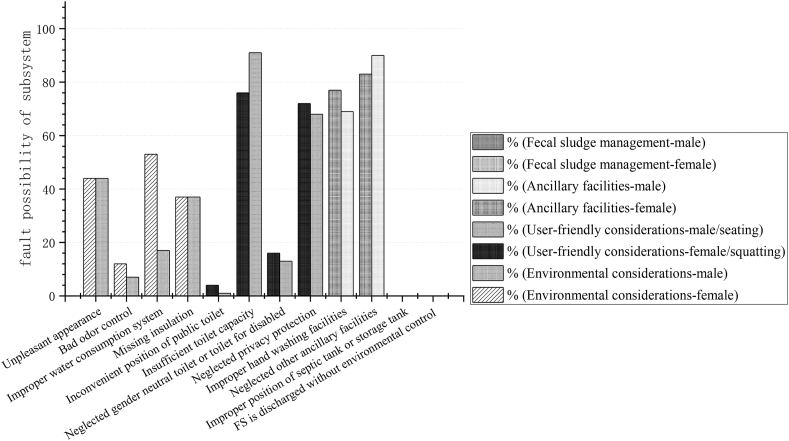
Total number of failures in various components of public toilet systems.

Fault tree for public toilet was structured to evaluate the potential risks as a result of the malfunction of various components. The total failure number of each type is summarized in Fig. 3.

### 3.1 Environmental considerations

Dirty public toilet is a health risk to users in urban settlements. The cleanliness is always prioritized to evaluate public toilets. Onsite investigators reported that 56% of male and female toilets are in good condition in terms of cleanliness. Approximately 44% of male and female toilets were unclean, including two female toilets, which caused severe discomfort to the inspector, and it can be treated as an undesirable event (failure event C1). Male toilet looks better than female toilets. This situation may be because women have many stringent hygiene requirements and feel subjectively uncomfortable with the same poor cleanliness condition. In addition to maintenance and cleaning, poor cleanliness condition in public toilets is also caused by people's consciousness on “shared resources”. People are less likely to temporarily value shared resources without paying. Community participation can contribute to the management success of sanitation systems (Davis et al., 2019).

Standard DB11/T 190–2016 provides that the minimum covered area for each pedestal pan/squatting pan is 2 m^3^. Most cubicles do not have comfortable spaces. We understand that no spare room is available for constructing new public toilets in Hutong neighborhoods. The architectural structure of Hutong leads to various layouts of Hutong public toilets, such as the long and narrow type, the encircling type, and the alternate permuted type. The reconstruction of public toilets in Hutong area proposed the special requirements of the plane design for the layout of as many toilet seats as possible under the premise of limited space and comfortable conditions.

Approximately 60% of public toilets were installed in the ventilation systems. In terms of odor control, 58% and 56% of male and female toilets, respectively, are satisfactory. However, 7% and 12% of male and female toilets, respectively, generate an unaccepted heavy odor, which can be treated as an undesirable event (failure event C2). Odor emission mainly results from dirty floor, unwashed sanitary fixtures, and remaining human excreta. If no proper ventilation is present, then heavy odor easily happens. However, 11 toilets without ventilation system still achieved satisfactory odor conditions because natural ventilation formed a good gas circulation inside and outside the toilet. A good natural ventilation structure could even replace the ventilation system during the planning and reconstruction of the toilet.

Flushing and tap water consumptions are intuitively judged by the investigator because the precise water flow test condition is limited during the field study. Water flow could be superfluous, moderate, or insufficient. Standard DB11/T 190–2016 provides that the flushing water volume for urinal and squatting pan per time is suggested to be less 1.5 and 4 L, respectively. Tap water should be water-saving. Approximately 31% of toilets are reported to adopt a proper water consumption system. Approximately 35% of toilets can be treated as failure (event C3) in terms of the water consumption system. One public toilet is still a dry toilet system without flushing water. Such unhealthful toilets imply that the public toilets in Hutong neighborhoods are not 100% covered by sanitary toilet; hence, improvement should be implemented. The surrounding residents reflected that some renovated public toilets only changed in the “appearance” instead of flushing volume reduction, and the flushing structure was even worse than before. No substantial grading change was observed before and after the renovation of the public toilet sanitation system. Understanding the reasons for the failure of sanitation systems is important to set the priority of limited resources. The high global sanitation system failure rate can be summed up in technical or nontechnical factors. The common characteristic of successful sanitation systems lied in sufficient municipal planning, adequate funding for operation, and management and community stakeholder's participation (Davis et al., 2019). The renovation of Hutong public toilets should fully investigate the demands of stakeholders, rationally allocate the flow of operation and management funds, and give priority to the introduction of plug-and-play technologies, such as foaming flushing toilet and recycled wastewater flushing toilet (China National Tourism Administration, 2017).

Local authorities in Beijing set the basic criteria to be “not cold in winter, not hot in summer, no odor” for toilet retrofitting in Hutong neighborhoods. Standard DB11/T 190–2016 provides that room temperature should be over 12 °C in winter and less than 30 °C in summer if an air conditioner is installed. More than 60% public toilets are equipped with heating facilities or air conditioners. Air conditioners appear only in a first class public toilet of a tourist area periphery. The survey found that different public toilets in the same area have heating or air conditioning, but whether the equipment is on or not differed, thereby resulting in a different comfort level. The unified management of public toilets should be strengthened to reach a unified standard. Public toilets will continue to exist for a long time in Hutong areas where it is difficult to have private sanitation facilities. A comfortable toilet environment should be created. Low satisfaction users generally have a high willingness to pay for public toilets, and operators should explore the potential market for building and maintaining high-quality public toilet facilities based on this concept (Mariwah et al., 2017). Air conditioning was running when nobody had used the toilet for a long time. An environmentally friendly sanitation system should be energy-saving and low-carbon. Thus, a trade-off exists between maintaining a reasonable temperature range and meeting low-carbon requirements.

### 3.2 User-friendly considerations

Public toilets should be located at the place where people traditionally and intuitively expect them to be; accordingly, people must be able to readily find them. Adequate signage is necessary to show where the public toilets are and their location. Signage of public toilets is normally found at the entrance of Hutong to indicate the direction of public toilets. Most public toilets are distributed on both sides of the street along the Hutong. However, some public toilets are placed at the back of the street, which are hard to be noticed. Only four public toilets visited are not readily found due to lack of signage. Given that most users are nearby residents, who are familiar with the surroundings, the absence and inconspicuousness of signage may only bring trouble to pedestrians and tourists.

An adequate number and distribution of public toilets that provides improved sanitation could improve the quality of life. Standard DB11/T 190–2016 provides that distance between public toilets should be 300–500 m for residential area, and each pedestal/squatting pan should serve a minimum capacity of 25–30 persons/day depending on local resident population density. The number of public toilets is unevenly distributed. The staff in the management room reported that only one public toilet of the three nearby Hutongs was available, and local people reflected the queue issue during the rush hour in the morning, even if improvement took place in recent years. Public toilets are an important part of building a livable and sustainable urban space (Afacan and Gurel, 2015). A reasonable number of public toilets in open space can reduce the negative psychological influence on pregnant women, the elderly, and incontinence patients and promote their timely self-health management (Bliss and Park, 2019). The location problem of Hutong public toilets is a location-allocation problem. This problem must be addressed to improve the effective use of resources and maximize service areas. Municipal departments need to proposed reasonable location selection planning before the construction or reconstruction to alleviate the congestion phenomenon of Hutong residents (Zhang et al., 2018).

Women are provided with half as many toilet facilities as men, thereby resulting in queues for women due to the long use time. The public toilet standard was revised in 2016 and increased the ratio of women to men toilet to 3:2 at the minimum. Approximately 34% of female toilets have 1–2 more squatting pans than male toilet, while only 9% of female toilets have pedestal pans, thereby indicating a huge gap to be filled against gender imbalance. The unchanged ratio of women to men toilet may be because of these toilets that had been newly built or rebuilt before the new standard was applied into practice. Hygiene and comfort are the basic requirements to sanitation facilities. Comfort includes not only sensory perception and mobility but also spiritual care, such as dignity, privacy, and fairness (UNICEF et al., 2018). Sustainable Development Goal (SDG) 6 calls for “adequate and equitable” sanitation facilities for all, with “special attention” to women, girls, and vulnerable groups, thereby ensuring that shared facilities and public toilets are well managed and “female friendly”. Gender equity in sanitation facilities, especially shared sanitation, is an inevitable topic. Women need a safe and comfortable place to replace menstrual supplies, sense of security, and protection, which needs long and a substantial amount of time. Improving the ratio of female to male cubicles can be the first step to facilitate the female inclusiveness; beyond that, higher requirements for public toilet were proposed in addition to affordability, availability, and safety (Hyun et al., 2019).

Gender-neutral toilet is considered to symbolize the development of humanization in terms of public toilet. Gender-neutral toilet normally serves more for than one person with different genders who can be present together. For instance, people can help their young children, older parents, or the disabled in a gender-neutral toilet. A gender-neutral toilet is also called family toilet or third-sex toilet. This type of toilet is common in public toilets in the tourist areas. In the survey, only 16 public toilets in Hutong neighborhoods deploy a separated gender-neutral toilet. More than 80% of public toilets were installed with pedestal/squatting pans with assistant equipment for disabled individuals.

Less than 10% public toilets were installed with pedestal pans (limited by one set only), and most pedestal pans were broken. On the one hand, pedestal pans are not well accepted by local people because they think that a pedestal pan is not as healthy as a squatting pan. Moreover, squatting pans could reduce the time spent in queues. Leading by cultural differences, seating toilets are generally confined to private toilets in China differentiated with “western” seated toilets (Agarwal and Jain, 2018). On the other hand, older people claimed the demand for pedestal pans, which could bring convenience for them. Local authorities must make a trade-off between hygiene issue and old people's demand. Maintenance can be a useful solution, but it needs a smart strategy. The case study on Public Toilet Map in the UK found that the public services could be improved through the disclosure and reuse of data from public sectors (Bichard and Knight, 2012). The real-time information of Hutong public toilets in nearby areas could be connected and shared to provide timely toilet seat allocation services for the elderly and patients.

Privacy was always neglected in the public toilets in China in the early days due to weak consciousness. In many developing countries, privacy is one of sanitation-related psychosocial stressors during routine sanitation practices (Sahoo et al., 2015). The study has also concluded that good latrine privacy is frequently associated with high latrine use (Garn et al., 2017). A minimum height of 1.8 m is required for toilet partition according to the standard. Approximately 27% of public toilets have no partition or a wall between the pedestal/squatting pan at all. Moreover, 43% of public toilets installed cannot meet the height standard. China fall behind western countries in terms of privacy. Chinese people have the habit of talking between neighborhoods when defecating if public toilets have no partition. Nowadays, awareness of sanitation practice behaviors has been improved with the progress of toilet revolution, and such “defecation culture” is considered an unhealthy behavior.

### 3.3 Ancillary facilities

Handwashing with soap is one of the key hygiene targets from SDG and is the only hygiene target monitored by WHO/UNICEF (Mara and Evans, 2018). A report indicated that handwashing is of extreme importance because the global disability adjusted life year (DALY) loss is due to “no access to handwashing facility” of 35.3 million years in 2016 for sexes and all ages (IHME, 2017). However, only 30% of public toilets in the survey are equipped with handwashing facility (water basin, soap/washing liquid, and hand dryer). Considering that most public toilets are close to users' home, this situation may increase the number of people who have access to washing facilities convenient to their usual living place rather than public toilet and consequently increase the frequency of post-defecation handwashing at home. Dirty hands increase the risk of gastrointestinal, respiratory, and skin infections. Inadequate hand washing time would result in prolonged bacteria carrying time. Hand hygiene is also one of the simplest and effective ways to stop the spread of a novel coronavirus during a COVID-19 pandemic (WHO and UNICEF, 2020). An observation on hand washing habits in a shopping center in Poland revealed that the average time to wash hands in public toilets is only 9 s, which neither meets the demand of cleanliness nor significantly reduces the bacterial flora carried after using the toilet (Malinowska-Borowska et al., 2015). Some people may forget to wash hands because of the space gap between the toilet and hand washing. Adults are likely to actively wash their hands than adolescents, and a healthy hand washing habit is critical to children's health. The hand-washing facilities in Hutong public toilets not only provide convenience for nonresidents but also help develop a good hand-washing reflex of residents, especially children. The possibility of fecal-oral transmission is still uncertain as COVID-19 spreads worldwide in 2020. Timely hand-washing after using the toilet and staying outdoors is one of the basic and effective means to combat the spread of the virus. Another interesting study in Ghana have shown that hand-washing as a toilet cleaning service increases residents' willingness to pay (Peprah et al., 2015).

Other ancillary facilities could offer convenience to users. More than 80% public toilets are equipped with cubicles hook for hanging personal belongings and handrail for sitting and standing. Only 20% of public toilets considered waste toilet paper bin according to oriental defecation habit. In western countries, such as United Kingdom, Germany, and Sweden, waste toilet paper bin does not exist in the public toilets. In Hutong areas where no waste toilet paper bins are present, investigators found that the improper structure of pipes made the paper float up after being flushed, thereby resulting in an uncomfortable sensation, which was also complained by nearby residents. Western countries are accustomed to flush human excreta with a decomposable toilet paper into a well-developed sewer system. However, the vast majority of Chinese still keep the habit of collecting waste toilet paper into a waste bin. Menstrual waste (mainly disposable sanitary napkins) disposal is particularly absent in the public toilets if no collection bin is present. In China, menstrual hygiene management is a neglected sanitation topic due to sociocultural beliefs, while studies on menstrual hygiene and waste management have been implemented in the least developed countries, such as Malawi (Roxburgh et al., 2020) and Zambia (Chinyama et al., 2018). The value of three menstrual products (disposable tampons and sanitary pads and reusable menstrual cups) were evaluated through a comparative life cycle assessment (Hait and Powers, 2019). Such interesting studies are still missing in China and would be worth of investigations in the future, on the condition that menstrual hygiene management is not a persistent taboo any more. The use of menstrual products is linked with menstrual waste. Without a bin, the menstrual waste would be thrown into the toilet and flushed away with human excreta. Inappropriate disposal of menstrual sanitary products and blockages is still a major problem in the water industry (Hawkins et al., 2019).

Only a few public toilets near tourist attractions installed mirror. Approximately 21.08% of public toilet missed floor drain. In such a case, cleaning staffs flush cleaning wastewater to squatting pan, thereby resulting in the enhancement of the daily waste load into the septic tank. Consequently, a frequent desludging of FS is required at a high operating cost.

### 3.4 FS management

The FS management components are composed of emptying, collection, transport, treatment, and end-use or disposal of FS(Bassan et al., 2014). “Safe feces disposal” in a public toilet is defined as defecation or disposal of feces (i.e. a sanitation option that allows human excreta to be isolated from the human living environment). A sewer system, which separates human excreta by a piping network, is the symbol of modern sanitation (Ushijima et al., 2014). Eight out of ten people with sewer connections live in urban areas (UNICEF and WHO, 2019). However, the sewer system is absent in Hutong neighborhoods for the sake of historical city planning and cultural heritage protection. In 2018, the collection and treatment amounts of FS from public toilets in urban Beijing are 2.01 and 1.95 million tons, respectively, with a treatment rate of 97.10% (Beijing Municipal Bureau of Statistics, 2019). Fig. 1 shows that the collected FS in urban Beijing has slightly fluctuated in the past decade. However, the amount of FS in urban China has greatly contemporaneously decreased as a result of the increased municipal pipelines that transport a substantial amount of human feces into sewage treatment plants (Cheng et al., 2018). Human feces from public toilets in Hutong neighborhoods cannot be connected to the sewer and municipal pipelines. This situation inevitably required extra human and material resources to collect the FS. The survey observed that FS from all visited public toilets were safely managed. Septic tanks or FS storage tanks are properly placed, and the underground water pollution is not expected. These phenomena indicate that the failure number of event B4 (C11 and C12) is zero. The FS must be strictly managed because all visited public toilets are located in the downtown and tourism areas of Beijing. The well-managed FS are due to puissant governmental control, which arranges well-organized desludging and transportation. Accordingly, all FS are managed well on the basis of our field survey. However, this notion does not mean that FS from public toilets can be managed well in other places of Beijing or China, let alone the developing world. Rural areas are susceptible to failure of FS management compared with urban areas (Berendes et al., 2017).

**Fig. 4:**
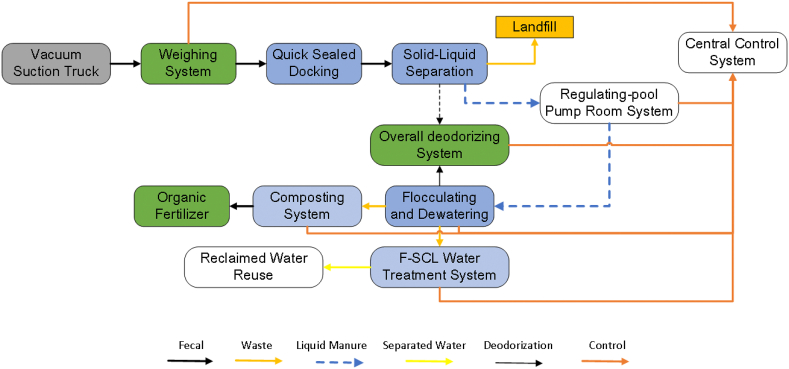
Typical FS treatment processes.

Along with the survey for public toilet, the investigators also visited some FS treatment plants in Beijing. The FS is sucked from the septic or storage tanks of public toilets and transported to the centralized FS treatment plant by the vacuum trucks. The FS is first weighted at the gate of the FS treatment plant. Then, the FS is unloaded in a sealed room, which is used for odor control and further processing. After the solid–liquid separation, the suspended substance and sediment of FS are removed. The solid part is collected and transported to the landfill, while the liquid part is pumped into the flocculation and dehydration system. The solid part (flocculent sludge) can be composted together with other organic matter (e.g., saw dust, greening waste, or cultivated waste). The liquid should be further treated as wastewater depending on the related standard and final destinations (discharge or reuse). The FS treatment plant is normally located near a wastewater treatment plant (WWTP) to easily discharge the effluent from the FS treatment plant into the WWTP. Fig. 4 shows a typical FS treatment process. The existing centralized FS treatment plant is regarded as the mature paradigm for most urban areas in the developing countries (Dodane et al., 2012). This mode also needs to be maintained at a cost of high resource consumption (e.g., trucks for transporting FS, land occupation, manpower to maintain, and flocculating agent to remove water from FS). Beijing is one of the extreme water-shortage cities in China. Water-consumption flushing toilets and resource-consumption FS collection are actually challenging to build a society of water conservation and resource recycling for Beijing. The public toilets in Hutong neighborhoods are a type of non-sewed sanitation systems. Toilets may be designed to allow the separation of urine and feces according to ISO 24521 (ISO, 2016). With the passing of ISO 30500 (ISO, 2018) and ISO 31800 (ISO, 2020), which aim at non-sewered sanitation systems and FS treatment units, respectively, many technical options for resource recovery from FS are considered onsite by stakeholders in developing countries, notably in non-sewered and off-grid environments. ISO30500, ISO31800, and ISO24251 proposed qualitative and quantitative requirements on the construction, maintenance, personnel training, health management, resource recovery, and online monitoring of onsite sanitation facilities and FS management without sewers. Many environment-friendly public toilets and FS management modes are urgently required to fight for the resource-oriented society.

Resource-oriented sanitation, also known as postmodern sanitation (Ushijima et al., 2014), is prioritized by all nations to optimize the sanitation value chain that creates and adds value to human excreta. Hence, future sanitation investments must incorporate the FS management to achieve safe excreta management, create added value for society while protecting human and ecosystem health, and promote sustainable development of urban environmental hygiene (Andersson et al., 2016). Studies have shown that the potential market-value products from FS include dry sludge as a fuel for combustion, protein derived from sludge processing as animal feed, sludge as a component in building materials, biogas/biomethane, biochar, biodiesel, and compost from co-treatment of FS, and kitchen waste/organic waste as clean energy, biofuel, and soil conditioner (Cheng et al., 2017; Diener et al., 2014; Krueger et al., 2020; Singh et al., 2017).

### 3.5 Calculations of fault probability

Based on the aforementioned statistical analysis, the fault probability of event B1 (P_B1_) is calculated on the basis of the fault probabilities of events C1, C2, C3 and C4, as follows:

0.79 P_B1_ = 1−(1−P_C1_)(1−P_C2_)(1−P_C3_)(1−P_C4_) = 1−(1–0.44)(1–0.095)(1–0.35)(1–0.37) ≈

Fault probability of event B2 (P_B2_) is based on failure number of events C5, C6, C7, C8, as follows:

0.96 P_B2_ = 1−(1−P_C5_)(1−P_C6_)(1−P_C7_)(1−P_C8_) = 1−(1–0.025)(1–0.835)(1–0.145)(1–0.7) ≈

Fault probability of event B3 (P_B3_) is based on failure numbers of events C9 and C10, so P_B3_ = 1-(1–0.73)(1–0.865) ≈0.96.

Fault probability of event B4 (P_B4_) is 0 based on the number of statistical failures of event C11 and C12, that is, 0, against 100 samples.

### 3.6 Determination of subsystem weight and calculation

The concept of Delphi method is employed to assist in recording failure scenarios and valuing the weight of four subsystems (Cheng et al., 2014). The Delphi method is a tool for gaining expert opinion to support, challenge, conclude, and test consensus. With regard to the sanitation sector, the Delphi method survey was once adopted to identify expert consensus on why and how to integrate sewered and non-sewered sanitation services into other basic urban services to achieve better sanitation and broad developmental outcomes (Scott et al., 2019). In our study, ten independent experts in this field of environmental sanitation were invited to present the weight of each subsystem according to their experience and cognitions, given that the whole weight of public toilet is one. Table 2 presents the scenarios of values. The weight provided by ten experts for four subsystems of public toilet differs from each other. This condition may be due to the different backgrounds and knowledge distinctions of the invited sanitation experts. For instance, some experts, who are prone to social research, may give priority to user-friendly considerations. By contrast, the expert with an environmental engineering background may highlight the back-end FS management and environmental considerations. The weight divergences among different experts are understandable and acceptable. The mean of each subsystem from ten experts is calculated and taken as the final weight.

**Table 2:** Weight for four subsystems of public toilet.

Subsystem	Environmental considerations (W_1_)	User-friendly considerations (W_2_)	Ancillary facilities (W_3_)	FS management (W_4_)	Public toilet
Expert 1	0.3	0.2	0.2	0.3	1
Expert 2	0.4	0.3	0.15	0.15	1
Expert 3	0.4	0.3	0.1	0.2	1
Expert 4	0.05	0.5	0.3	0.15	1
Expert 5	0.3	0.3	0.1	0.3	1
Expert 6	0.25	0.25	0.25	0.25	1
Expert 7	0.3	0.3	0.2	0.2	1
Expert 8	0.2	0.3	0.3	0.2	1
Expert 9	0.2	0.2	0.2	0.4	1
Expert 10	0.25	0.25	0.25	0.25	1
Mean	0.265	0.29	0.205	0.24	1

The fault probability of top event A (P_A_), which stands for the abnormal operation of public toilet, is calculated on the basis of the weight:

P_A_= P_B1_*W_1_ + P_B2_*W_2_ + P_B3_*W_3_ + P_B4_*W_4_ = 0.79*0.265 + 0.96*0.29 + 0.96*0.205 + 0*0.0.24 = 0.6846≈0.68

This result can be adopted to value the fault probability of the public toilet on the basis of the probability theory and reflect the abnormal operating status of public toilets in Hutong neighborhoods of Beijing. The well-operation ratio is approximately 32%, thereby implying that the operating status of public toilets is unsatisfying. Previous studies have mentioned that the constraints concerning the good operation of public toilets exist at the planning rationality, investment allocation, and stakeholder participation. If in planning, user demands are emphasized, and some technical problems are overcome, then further improvement of the well-operation ratio is possible.

## 4 Conclusions

Public toilets are the predominant form of sanitation for people who dwell in high-density Hutong neighborhoods in downtown Beijing. Public toilets are of great importance in contributing to the health and well-being of the society. Beijing Hutong is a representative of the special historical culture. The public toilet in Hutong neighborhoods should have profound historical connotation and advanced cultural ideology. From the perspectives of environmental consideration, user-friendly consideration, ancillary facilities, and FS management, the barriers toward a decent and environment-friendly public toilet in the Hutong neighborhoods of Beijing are identified on the basis of the field survey covering a sample volume of 100 public toilets. Four subsystems of public toilets are defined and depicted using a fault tree approach. The statistical analysis of targeted cases indicated that the fault probabilities of environmental considerations, user-friendly considerations, ancillary facilities, and FS management are calculated as 0.79, 0.96, 0.96 and 0, respectively. We weighted the four subsystems by using a Delphi method. The well-operation ratio of public toilets in the Hutong neighborhoods of Beijing is calculated as 32%, thereby indicating an unsatisfying result. In addition, the sanitation service value chain can be further optimized, in light of water-saving toilet and value-added by-products from FS. Future research could focus on menstrual hygiene management, hand hygiene behavior, willing-to-pay mechanism, and trade-off between natural ventilation and insulation system inside a public toilet.

## Author contribution

Rui Yan: Writing – original draft, Visualization, Investigation, Shikun Cheng: Conceptualization, Methodology, Writing – review & editing, Jingang Chen: Investigation, Data curation, Writing – review & editing, Xiangkai Li: Investigation, Data curation, Writing – review & editing, Sumit Sharma: Investigation, Data curation, Sayed Mohammad Nazim Uddin: Writing – review & editing, Heinz-Peter Mang: Writing – review & editing, Cong Chen: Writing – review & editing, Zifu Li: Funding acquisition, Tianxin Li: Funding acquisition, Xuemei Wang: Writing – review & editing, Supervision

## Declaration of competing interest

The authors declare that they have no known competing financial interests or personal relationships that could have appeared to influence the work reported in this paper.

## Footnotes

1 Hutong is originally a Mongol word that refers to the space between tents or the way to the well.
