# Supplementary material for: Operating status of public toilets in the Hutong neighborhoods of Beijing: An empirical study
Source: J Environ Manage. 2021 Jun 1;287:112252. doi: 10.1016/j.jenvman.2021.112252 (PMC8075803; doi:10.1016/j.jenvman.2021.112252)
Supplement: Multimedia component 1 [file mmc1.doc]

**Operating status of public toilets in** **the Hutong neighborhoods of Beijing: an empirical study**

**Supplementary material - Photos about the public toilet and components, taken by the investigators (Rui Yan, Jingang Chen, Xiangkai Li)**


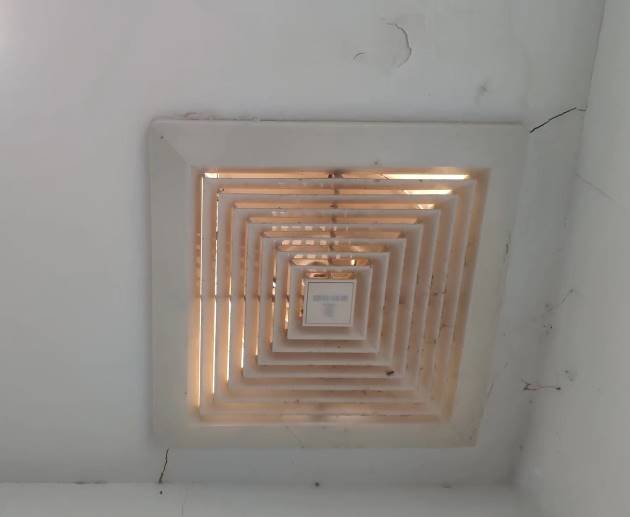


Fig. S1 Ventilation opening


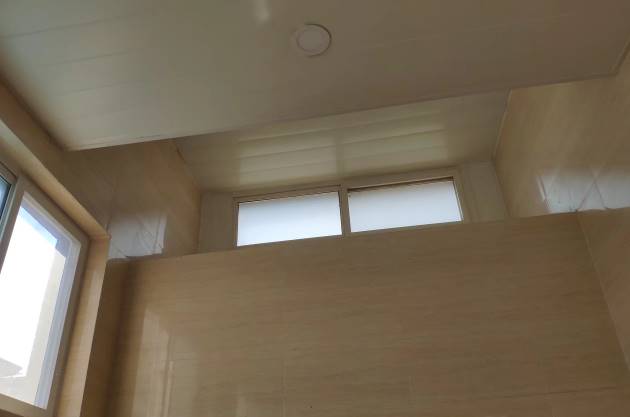


Fig. S2 Natural ventilation


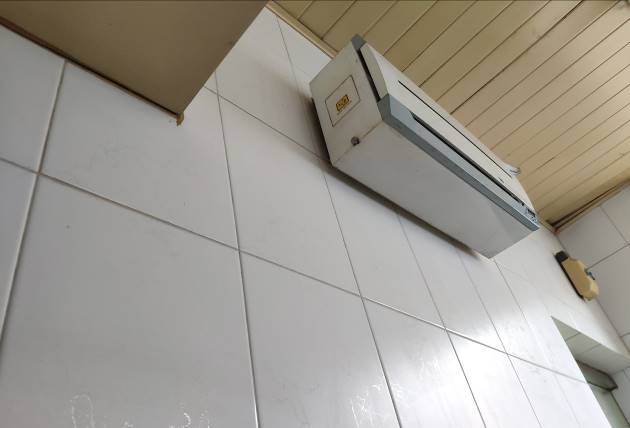


Fig. S3 Air conditioner


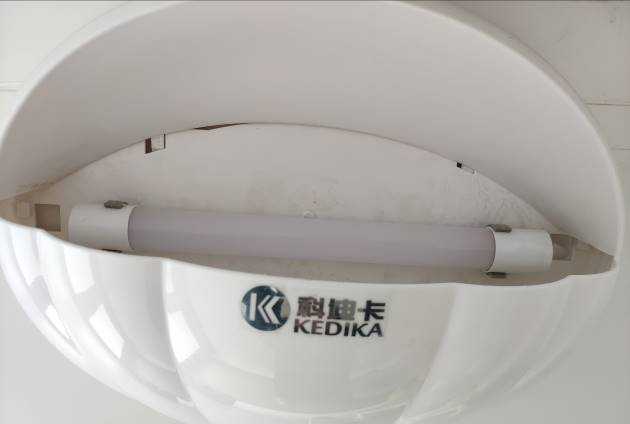


Fig. S4 Lighting on the wall


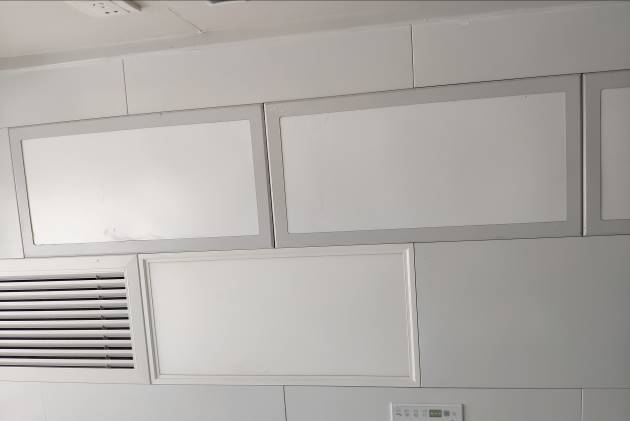


Fig. S5 Ceiling light


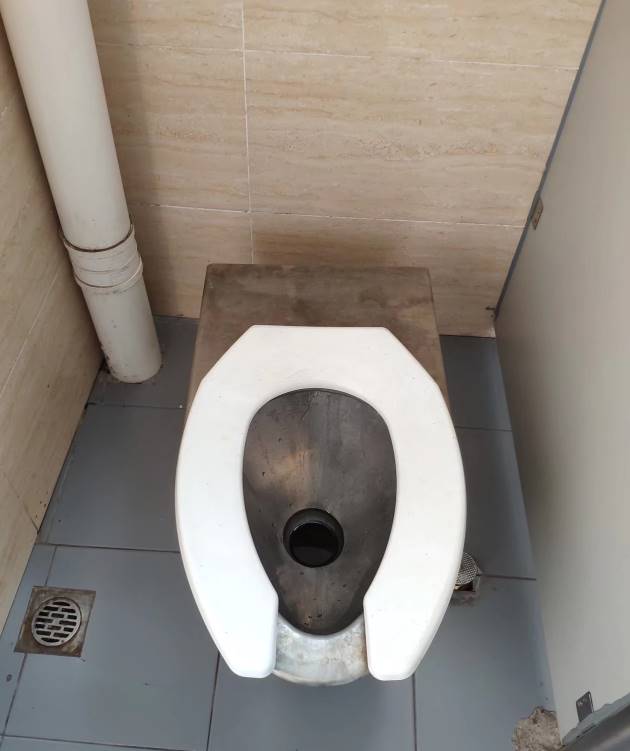


Fig. S6 Simple seating pan


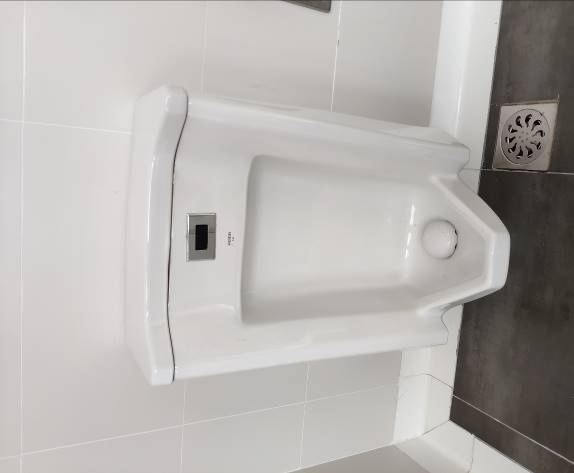


Fig. S7 Urinal for males


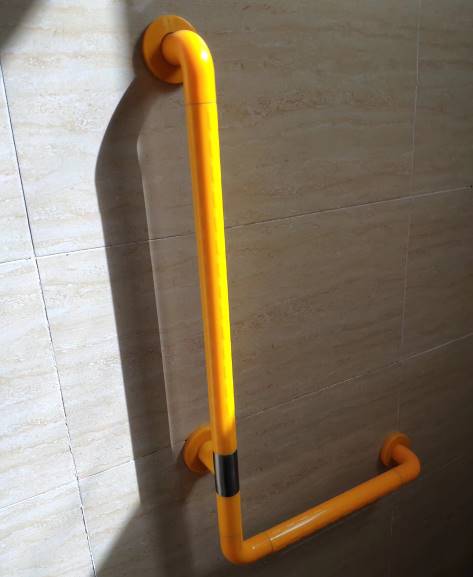

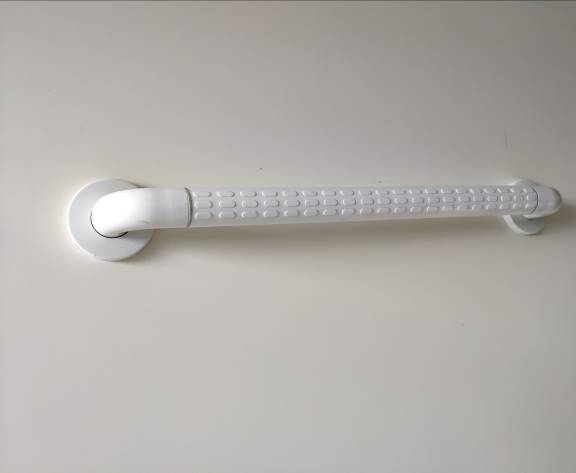


Fig. S8 Barrier-free handrail


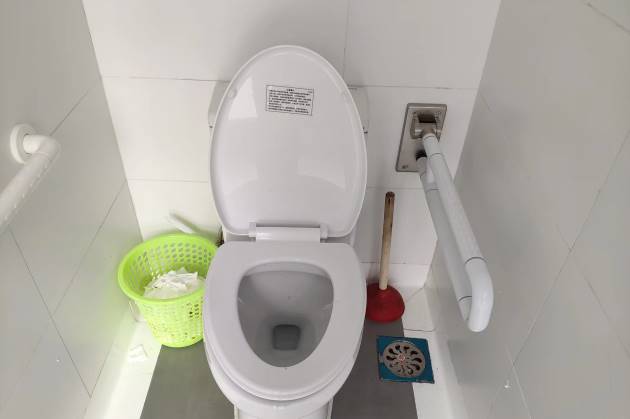


Fig. S9 Gender-neutral toilet


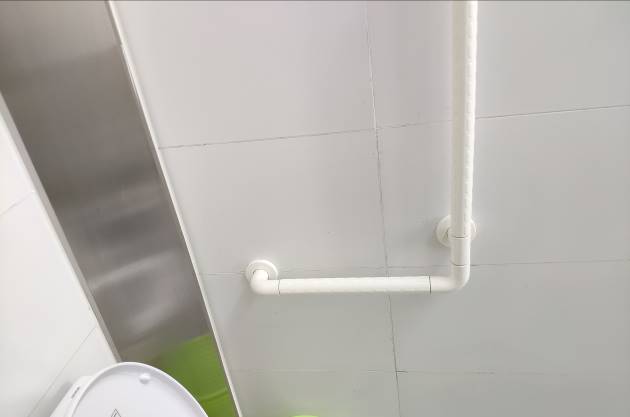


Fig. S10 Urinal or pedestal pan installed for disabled people or elders


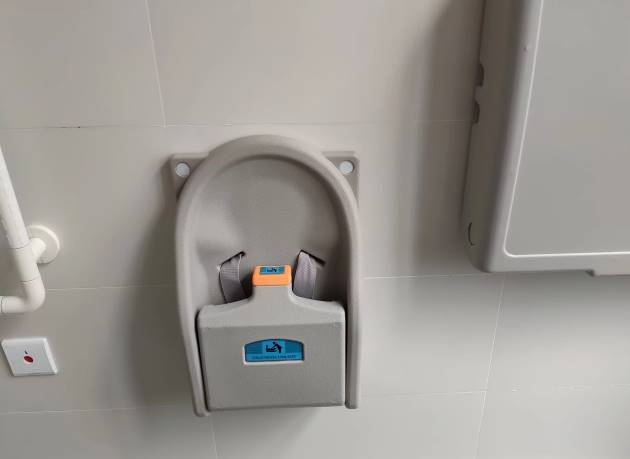


Fig. S11 Mothor-and-child facilities


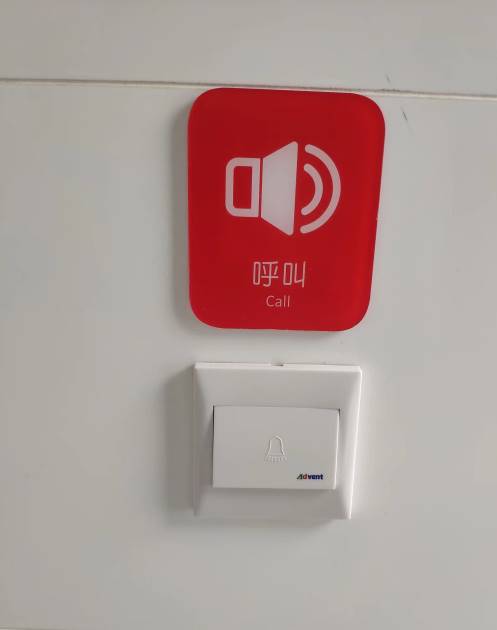


Fig. S12 Emergency bell


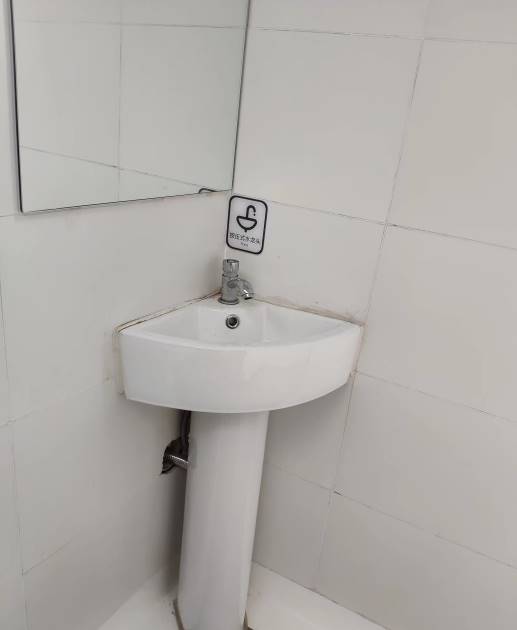


Fig. S13 Washing sink and mirror in the corner


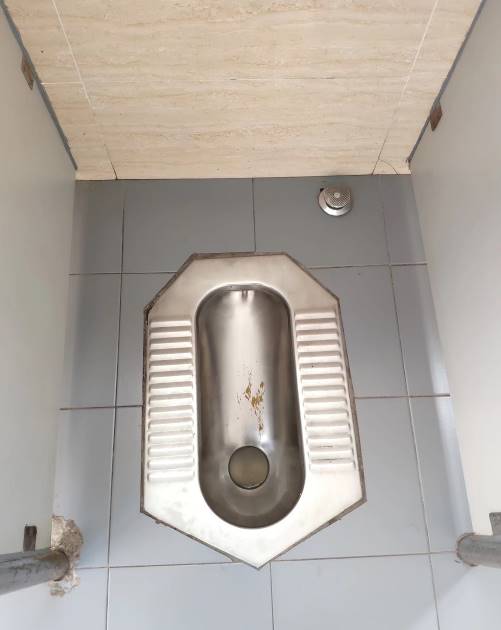


Fig. S14 lack of toilet paper bins


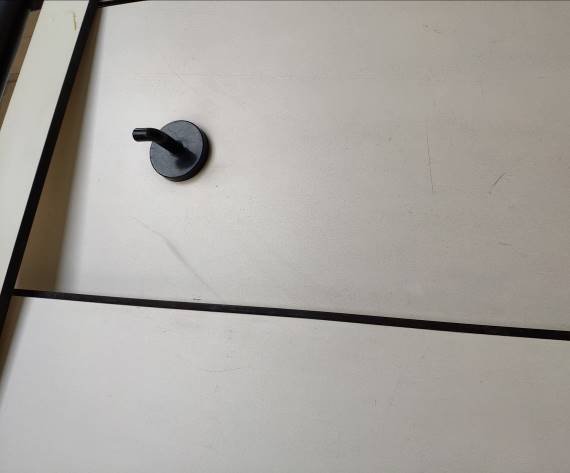


Fig. S15 Cubicle hook on the door


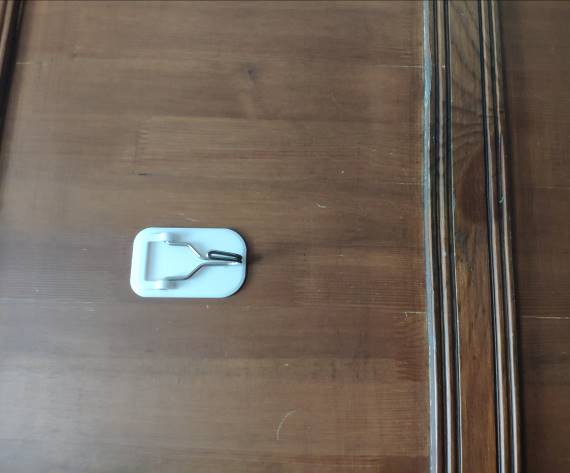


Fig. S16 Cubicle hook on the wall


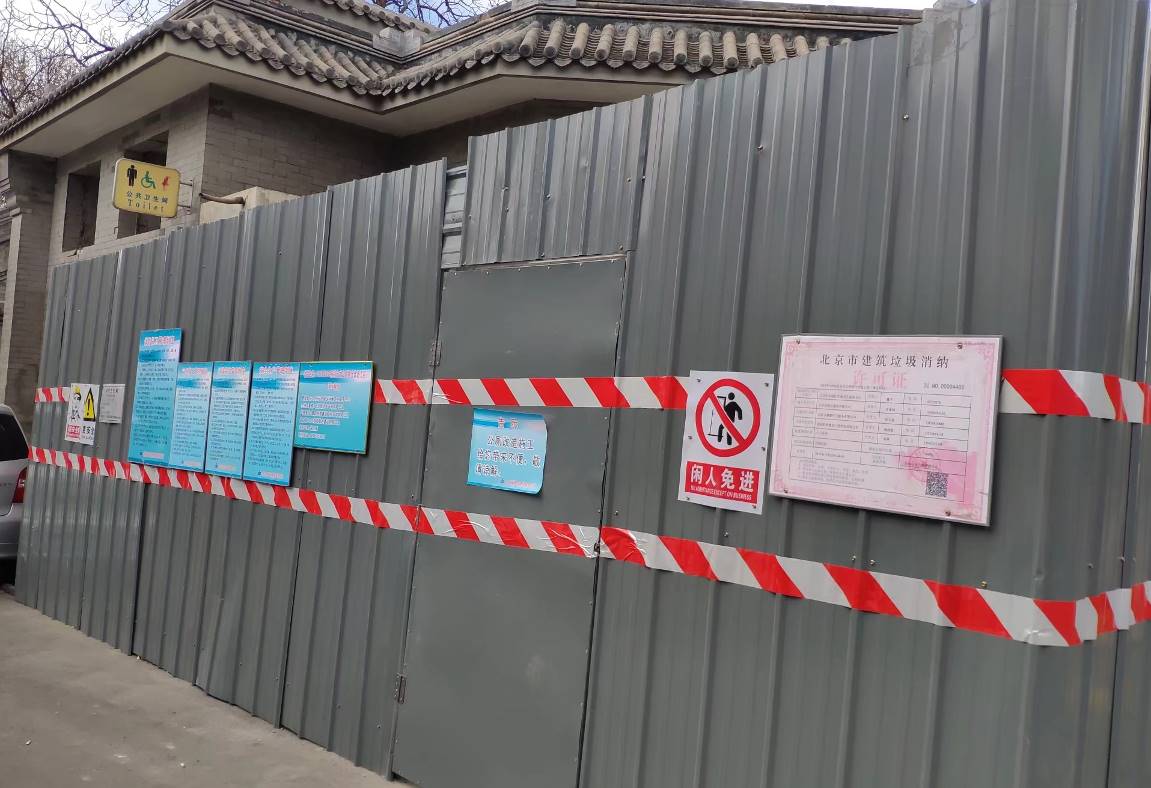


Fig. S17 Hutong public toilets renovating


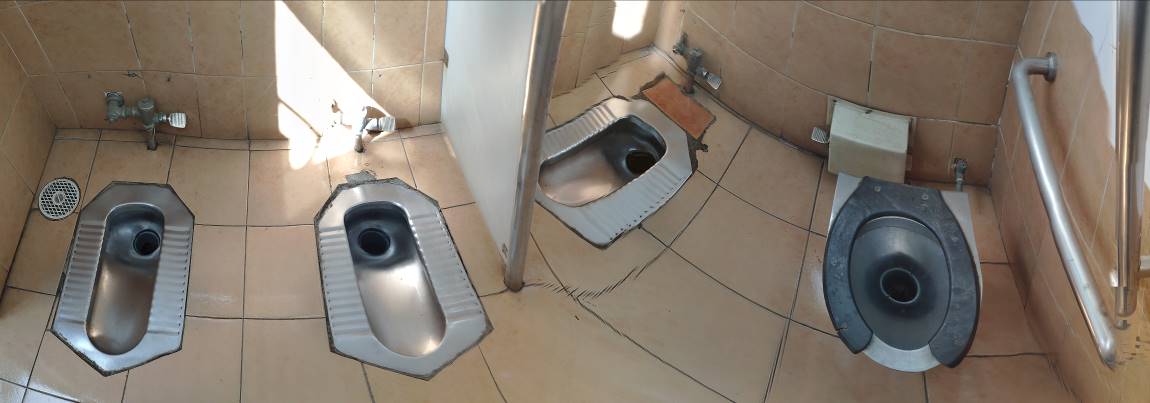


Fig. S18 A complete view of the unrenovated public toilet
